# A Case of Non-Germinomatous Germ Cell Tumors of the Frontal Lobe Arising From the Lateral Ventricle With a Synchronous Pineal Lesion

**DOI:** 10.7759/cureus.29895

**Published:** 2022-10-04

**Authors:** Vipin Kharade, Saikat Das, Arnav Tiwari, Manish Gupta

**Affiliations:** 1 Department of Radiotherapy, All India Institute of Medical Sciences, Bhopal, IND; 2 Department of Radiation Oncology, All India Institute of Medical Sciences, Bhopal, IND

**Keywords:** germinomas, irradiation, craniospinal, germ cell tumours, non-germinomatous

## Abstract

Non-germinomatous germ cell tumours (NGGCT) are rare intracranial tumours that account for 1% to 3% of cases. They are usually seen in the pineal and suprasellar regions. NGGCT of the frontal lobe arising from the lateral ventricle with a synchronous pineal lesion is uncommon. We present a case of NGGCT with multifocal lesions in the pineal gland, frontal lobe, and pons treated with chemotherapy followed by craniospinal irradiation (CSI).

## Introduction

Non-germinomatous germ cell tumours (NGGCT) are rare intracranial tumours occurring in adolescents and young adults. They are predominantly seen in the pineal and suprasellar regions and account for 1% to 3% of cases [1]. They are predominantly seen in the pineal and suprasellar regions [2]. The presentation in unusual areas like the frontal lobe is extremely rare with only a few documented case reports. NGGCT of the frontal lobe region with a lesion in the pineal region can be multi-focal primary or metastatic lesions [3]. Multifocal germ cell tumours are often empirically treated as germinomas. However, they should be treated as NGGCT if the alpha-fetoprotein (AFP) levels are elevated. We discuss the presentation, diagnosis, and treatment of an NGGCT with multifocal lesions in the frontal lobe, pineal region, and pons which was treated with chemotherapy and craniospinal irradiation (CSI).

## Case presentation

**Figure 1:**
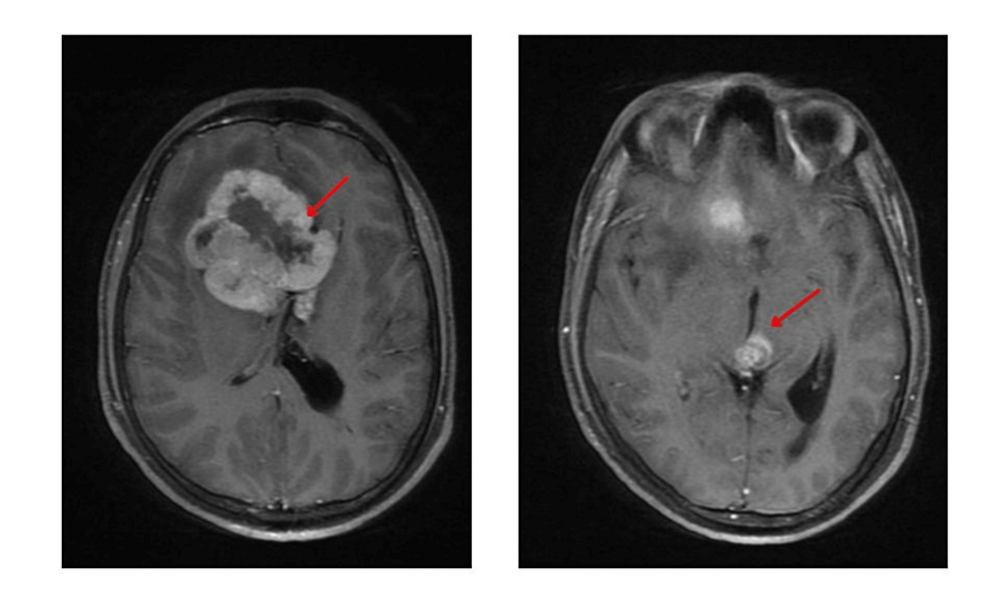
Pretreatment MRI showing the lesions

A 16-year-old male presented with complaints of global headache associated with projectile vomiting. It was not associated with any motor or sensory deficits, fever, neck stiffness, seizures, or loss of consciousness. The clinical findings were consistent with raised intracranial pressure. There were no clinical signs or laboratory findings of infection. The findings in the contrast-enhanced computed tomography (CECT) brain and magnetic resonance imaging (MRI) brain showed a frontal lobe lesion of size 6.0 x 5.5 x 4.0 cm within the frontal horn and anterior body of the right lateral ventricle which appeared as a multiloculated hyperdense lesion on CT and heterogeneously isointense with a hyperintense component and patchy areas of T2 hypointensity. On post-contrast MRI, the mass revealed intense heterogeneous enhancement with central non-enhancing areas suggestive of necrosis. There were areas of susceptibility weighted imaging (SWI) blooming within the lesion and correlation with CT was suggestive of haemorrhage (Figure 1). The mass was crossing midline, abutting and displacing septum pellucidum with associated hydrocephalus in the left lateral ventricle and involving the genu and anterior body of the corpus callosum (midline shift). Another lesion with similar characteristics of size 1.4 x 1.4 cm is seen in the pineal region involving the thalamus and midbrain. A tiny nodule of 4 mm showing hypointense signal intensity and homogenous post-contrast enhancement was seen on the posterior surface of the pons. These findings were suggestive of neoplastic mass lesions - probably germinomas. The serum beta human chorionic gonadotropin (HCG) and AFP levels were obtained which were 0.759 IU/L (<2 IU/L) and 26710 ng/mL (<10 ng/mL), respectively. The location of the tumour and increased chances of haemorrhage in germ cell tumours made biopsy a high-risk procedure and it was deferred after neurosurgical evaluation. The cerebrospinal fluid (CSF) examination was not done due to raised intracranial pressure causing a risk of herniation. Given the raised AFP levels, the patient was diagnosed with NGGCT with the possibility of a yolk sac tumour.

**Figure 2:**
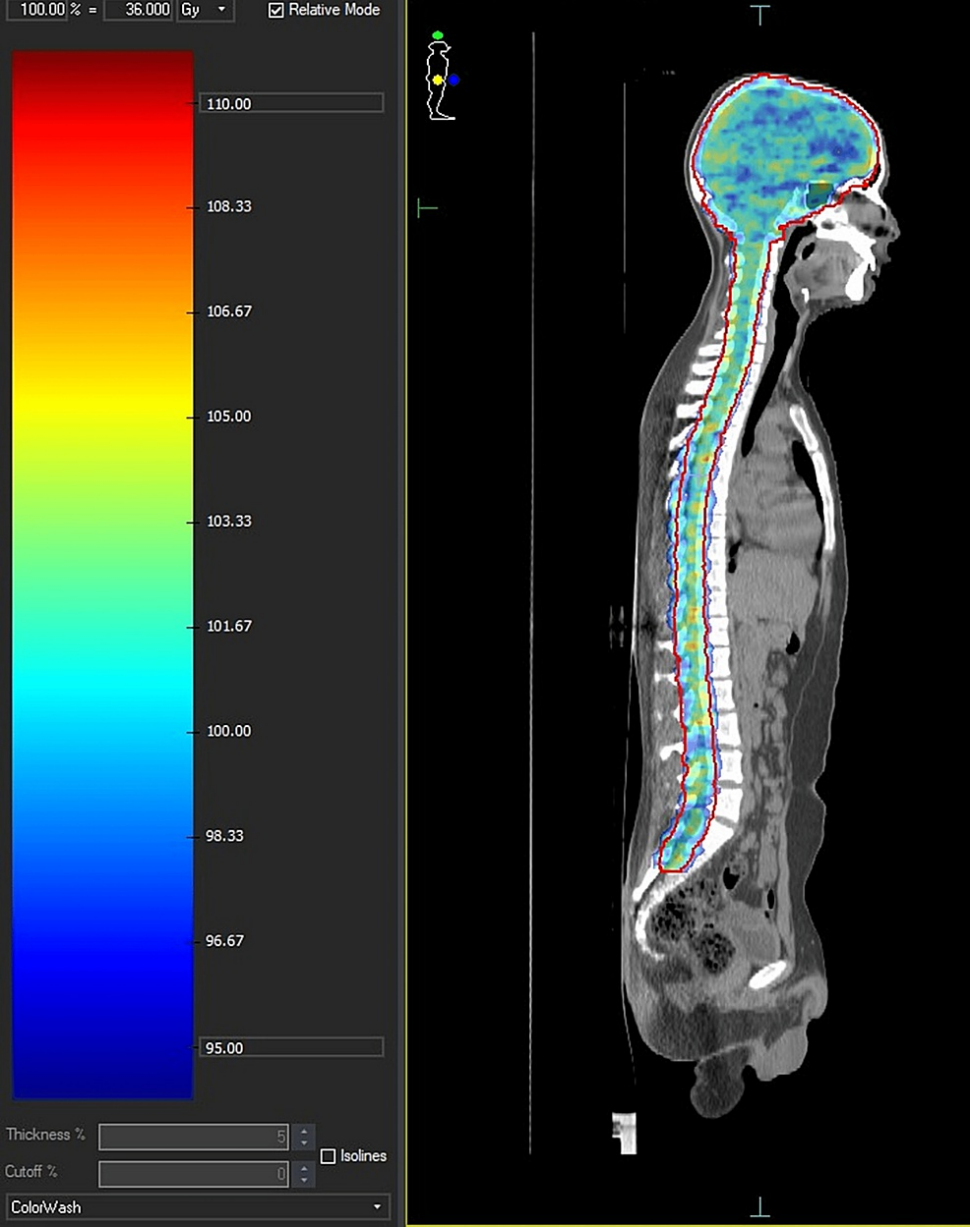
Craniospinal radiation showing dose distribution in color wash

**Figure 3:**
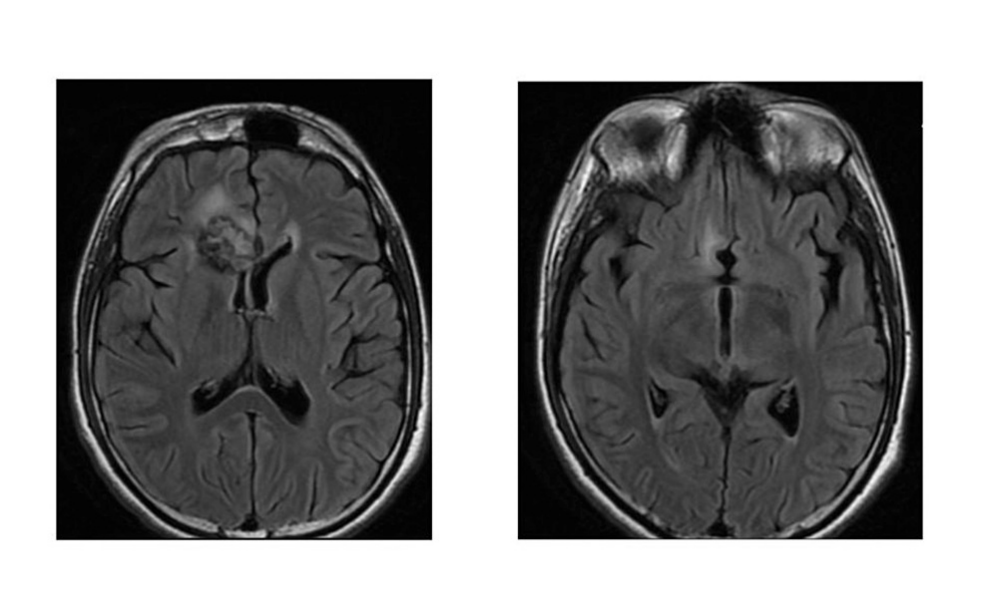
Post-treatment MRI imaging showing the response of the lesion

He was planned for chemotherapy with two cycles of CarboPEI regimen (carboplatin 600 mg/m2 on day 1, etoposide 100 mg/m2 on days 1,2,3,22,23,24, and ifosfamide 1800 mg/m2 on days 22-26, total two cycles) [4]. After two cycles, radiological and biochemical response assessment was done. Post-chemotherapy imaging showed a marked reduction in the size of the frontal lobe lesion with a resolution of features of raised intracranial pressure and the other two lesions. CSF was examined and was negative for atypical cells and AFP levels were within the normal range. There was a marked reduction in the serum AFP levels with the values decreasing to 19.7 IU/ml post chemotherapy. He was then planned for CSI treatment in the line of a non-germinomatous tumour; a phase I initial dose of 36 Gy in 20 fractions followed by 18 Gy in 10 fractions phase II boost to the gross disease (total dose 54 Gy) (Figure 2). His serum AFP levels were within normal limits post radiotherapy. Post the treatment imaging, the tumour had reduced in size (Figure 3). The patient is on follow up and the serum AFP levels are within normal limits after two years of completion of treatment.

## Discussion

Intracranial germ cell tumours are relatively rare tumours of the central nervous system. They often present as an isolated lesion of the pineal region or the suprasellar region. Involvement of sites other than these regions, with the exclusion of direct subependymal extension from these sites along the ventricle, is considered to represent metastatic disease [2]. In our case, as the frontal lobe lesion was seen within the ventricle, it was considered a primary synchronous with lesions in the pineal gland and the pons. Studies have shown that NGGCT appears isodense to hyperdense on CT with post-contrast enhancement. On MRI, they usually appear isointense to hyperintense, with localised hypointensity and heterogeneous enhancement [5]. Our findings were similar to those reported in the literature and suggested a diagnosis of germ cell tumour. Takahashi et al. have reported a case of an NGGCT with a frontal lobe lesion [6]. Often these synchronous lesions with a background of detectable b-HCG and normal to mildly raised AFP are empirically diagnosed as germinomatous germ cell tumours [7]. A biopsy is often omitted in these cases as they are said to be pathognomonic of germinomas and reports of synchronous non-germinomatous tumours are very limited. This approach is supported in the Children’s Oncology Group’s most recent protocol for intracranial GCT [4,8]. A diagnosis of NGGCT should be considered in the case of multifocal GCTs with increased AFP levels. The diagnosis should be confirmed with a biopsy if it is permissible in the clinical setting. In cases of synchronous lesions, it is critical to consider non-germinomatous tumours because the therapy and prognosis for germinomas and non-germinomas differ dramatically. NGGCTs often require a more aggressive approach. While germinomas can be treated only with radiotherapy, NGGCTs often require a combined approach for a better survival outcome. They are treated with high-dose chemotherapy followed by CSI [9].

Our patient had synchronous lesions of the pineal, frontal lobe, and pons. As the serum AFP levels were elevated, he was diagnosed with an NGGCT. He was then treated with high-dose chemotherapy followed by CSI. The patient is currently doing well with no radiological or biochemical progression.

## Conclusions

Multifocal GCTs should not always be empirically treated as germinomas. A diagnosis of NGGCT should be ruled out with tumour markers and biopsy. However, our case illustrates that biopsy may not be feasible in some situations due to raised intracranial pressure. Multi-disciplinary discussions and prompt institution of therapy are recommended. In our case, good disease control was achieved with chemotherapy and CSI; this illustrates the role of CSI in such situations.
